# Angiopoietin-1 and ανβ3 integrin peptide promote the therapeutic effects of L-serine in an amyotrophic lateral sclerosis/Parkinsonism dementia complex model

**DOI:** 10.18632/aging.101661

**Published:** 2018-11-25

**Authors:** Hua-ying Cai, Ke-wei Tian, Yuan-yuan Zhang, Hong Jiang, Shu Han

**Affiliations:** 1 Institute of Anatomy and Cell Biology, Medical College, Zhejiang University, Hangzhou, China; 2 Department of Neurology, Sir Run Run Shaw Hospital, Medical College, Zhejiang University, Hangzhou, China; 3 Department of Electrophysiology, Sir Run Run Shaw Hospital, Medical College, Zhejiang University, Hangzhou, China

**Keywords:** amyotrophic lateral sclerosis (ALS), amyotrophic lateral sclerosis/Parkinsonism dementia complex (ALS/PDC), beta-N-methylamino-L-alanine (L-BMAA), L-serine, C16, angiopoietin-1

## Abstract

Amyotrophic lateral sclerosis (ALS) is an adult disorder of neurodegeneration that manifests as the destruction of upper and lower motor neurons. Beta-N-methylamino-L-alanine (L-BMAA), an amino acid not present in proteins, was found to cause intraneuronal protein misfolding and to induce ALS/Parkinsonism dementia complex (PDC), which presents symptoms analogous to those of Alzheimer’s-like dementia and Parkinsonism. L-serine suppresses the erroneous incorporation of L-BMAA into proteins in the human nervous system. In this study, angiopoietin-1, an endothelial growth factor crucial for vascular development and angiogenesis, and the integrin αvβ3 binding peptide C16, which inhibits inflammatory cell infiltration, were utilized to improve the local microenvironment within the central nervous system of an ALS/PDC rodent model by minimizing inflammation. Our results revealed that L-serine application yielded better effects than C16+ angiopoietin-1 treatment alone for alleviating apoptotic and autophagic changes and improving cognition and electrophysiological dysfunction, but not for improving the inflammatory micro-environment in the central nerve system, while further advances in attenuating the functional disability and pathological impairment induced by L-BMAA could be achieved by co-treatment with C16 and angiopoietin-1 in addition to L-serine. Therefore, C16+ angiopoietin-1 could be beneficial as a supplement to promote the effects of L-serine treatment.

## Introduction

Amyotrophic lateral sclerosis (ALS) is an adult disease that manifests as gradual paralysis of muscles resulting from motor neuron degeneration. Only 5–10% of ALS cases are familial, whereas 90% of ALS cases are sporadic ALS [1]. Beta-N-methylamino-L-alanine (L-BMAA), an amino acid not derived from proteins, has been detected in the central nervous system of patients with ALS/Parkinsonism dementia complex (ALS/PDC), indicating that L-BMAA can traverse the blood–brain barrier in patients suffering from the disorder and implicating this amino acid in the development of neurodegenerative diseases [2-5]. Moreover, previous studies showed that L-BMAA can be selectively toxic to motor neurons, with a dose of 30 μM eliciting considerable cell death [3].

Inflammation is increasingly being recognized as a pathophysiological mechanism in neurodegenerative diseases. A common feature of neurodegenerative diseases including ALS/PDC is the presence of inflammation brought about by activated glial cells, primarily astrocytes and microglia, and T cells [6]. Patients with ALS have evidence of chronic inflammation demonstrated by infiltration of inflammatory macrophages [7]. Inflammatory cells from the blood invade the spinal cord and mediate production of a net neurotoxic non-neuronal milieu encompassing the motor neurons, ultimately causing the death of more motor neurons. Moreover, inflammatory macrophages may phagocytize apoptotic as well as non-apoptotic motor neurons, reactive microglial cells and astrocytes actively, also leading to the destruction of motoneurons [8].

Given that cells of the immune system, which have traversed the blood–brain barrier (BBB), may exert adverse effects on the endogenous micromilieu of the central nervous system (CNS), reducing their crossing of the BBB is important for maintaining a physiologically stable equilibrium of the endogenous micro-milieu in the CNS and for alleviating the pathological destruction of motor neurons. Angiopoietin-1 (Ang-1) is a member of the vascular endothelial growth factor family that binds to the receptor tyrosine kinase Tie2 to stabilize the endothelium and reduce endothelial permeability to permit normal cardiovascular development [9]. Another peptide, C16 (KAFDITYVRLKF), exhibits selective binding to integrin αvβ3 that is expressed on endothelial cells and competitively interferes with the binding between leukocytes and endothelial cells that is necessary for transmigration, thereby attenuating inflammation [10]. Previous research has demonstrated that treatment with C16 peptide in conjunction with Ang-1 further suppresses leukocyte/lymphocyte infiltration and microglia/macrophage activation [11,12]. Therefore, the present investigation aimed to assess the efficacy of C16 and Ang-1 in ameliorating inflammation within the local microenvironment in an ALS/PDC rodent model and to further test whether this treatment pairing could promote the therapeutic effect of L-serine, which has been reported to reduce the risk of neurodegeneration triggered by the environmental toxin L-BMAA [13].

## RESULTS

### L-BMAA elicited functional impairment and electrophysiological alterations, whereas C16+Ang-1, L-serine and the combined treatment reversed electrophysiological dysfunction and mitigated functional impairment

**Figure 1:**
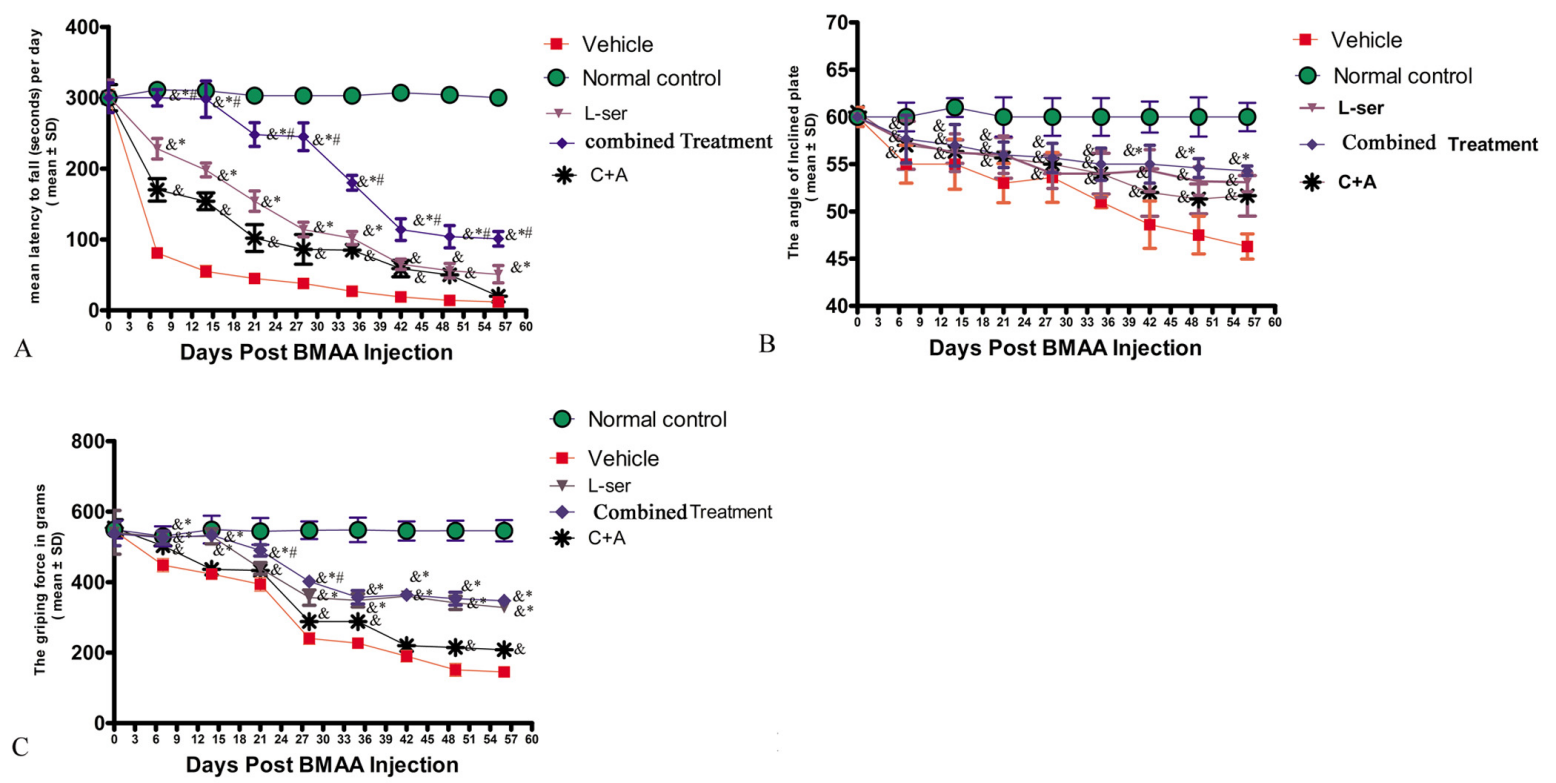
Motor functional evaluation revealed neurological impairments as early as 2 weeks after L-BMAA injection, and the C16+Ang-1 and L-serine treatments alleviated the severity of disabilities, with the combined treatment further improving motor function. (**A**) Time on the rod in the rotarod test. (**B**) Angle at which the rats fell in the inclined plane test. (**C**) Average grip strength of forepaws. ^&^ p<0.05 vs. the control group, *p<0.05 vs. the C16+Ang-1 group, and ^#^p<0.05 vs. the L-ser group.

Following L-BMAA injections, the model rats exhibited functional impairments relative to the normal controls. In the rotarod test (Fig. 1A), the model rats were unable to stay on the device as long as the normal control rats from 1–2 weeks post-injection (pi) (p<0.05). In addition, the tilt angles at which the model rats could remain standing was significantly smaller than that for the control rats (p<0.05, Fig. 1B). The mean grip strength of the forepaws of model rats was less than that of the control rats (p<0.05, Fig. 1C).

Importantly, the vehicle-treated model rats had considerably more severe functional impairments than model rats treated with C16+Ang-1 and L-serine, individually or in combination, with those receiving both treatments showing the greatest effect at each time point (Fig. 1A-C). The data indicated that L-BMAA treatment led to a marked reduction of muscle force both in forepaws and hind paws, which worsened with time, but could be reversed by C16+Ang1 and L-serine treatments, with the combined treatment yielding the best effects in improving motor function.

The electrophysiological findings revealed pathological alterations in nerve conduction. In the vehicle-treated model group, at each time point, the MEP amplitude (an index of the number of surviving fibers) was decreased, and the MEP latency (an index of the speed of conduction) was lengthened, in comparison with the normal controls. However, treatment with C16+Ang-1 increased the amplitude and reduced the latency, while L-serine treatment and the combined treatment produced even more pronounced effects (Table 1). In addition, in the vehicle-treated model group, the mean amplitude of MUPs was elevated, the mean duration increased, and the proportion of polyphasic potentials heightened, signifying loss of muscular innervations, and treatment with C16+Ang-1, L-serine, or both significantly reversed these phenomena (Table 2).

**Table 1:** L-BMAA administration prolonged motor-evoked potential (MEP) latency and lowered MEP amplitude.

Time point	Group	Latency (ms)	Wave amplitude (μV)
2 weeks	Normal	2.1±0.5**	3.6±0.21**
	Vehicle	6.6±0.31	0.2±0.16
	C+A	5.35±0.28*	1.2±0.25**
	L-ser	4.26±0.3**	2.26±0.11**
	Combined	4.9±0.55**	3.7±0.21**
4 weeks	Vehicle	7.1±0.65	0.2±0.18
	C+A	4.6±0.42**	3.0±0.78**
	L-ser	4.22±0.10**	2.2±0.42**
	Combined	4.19±0.68**	4.6±0.43**
8 weeks	Vehicle	7.3±0.5	0.2±0.08
	C+A	2.3±0.12**	2.0±0.28*
	L-ser	4.2±0.9**	3.67±0.32**
	Combined	4.35±0.18**	4.75±0.55**

C+A, C16+Ang-1; L-ser, L-serine; combined, C16+Ang-1 and L-serine. Data are expressed as mean±SD, n=5. *p<0.05 and **p<0.01 *vs.* the vehicle group (one-way ANOVA).

**Table 2:** L-BMAA administration increased the amplitude, duration, and rate of polyphasic action potential of motor unit potentials.

Time point	Group	Wave amplitude (μV)	Duration (ms)	Polyphasic action potential (%)
2 weeks	Normal	412±10.22**	10.12±4.35**	7±1.12%**
	Vehicle	1012±31.58	20.6±2.69	30±12%
	C+A	766±20.14**	17.6±3.68*	23±2.58%**
	L-ser	606±40.88**	13.9±4.15**	19±2.26%**
	Combined	523±28.64**	9.1±2.28**	17±2.32%**
4 weeks	Vehicle	1000±30.88	23.6±3.2	50±7.98%
	C+A	756±25.64**	16.6±3.25**	29±6.88%**
	L-ser	535±25.8**	14.7±1.16**	14.4±1.96%**
	Combined	471±9.4**	9.7±2.34**	10±3.54%**
8 weeks	Vehicle	986±9.4	22.6±4.5	50±7.5%
	C+A	560±28.9**	17.9±1.15**	27±3.55%**
	L-ser	492±30.46**	12.7±2.85**	20±2.16%**
	Combined	470±15.85**	10.9±2.88**	5±0.28%**

C+A, C16+Ang-1; L-ser, L-serine; combined, C16+Ang-1 and L-serine. Data are expressed as mean±SD, n=5. *p<0.05 and **p<0.01 *vs.* the control group (one-way ANOVA).

In the object–place recognition task, the vehicle-treated model rats spent almost the same time scrutinizing novel and familiar juveniles during the test, concordant with failure of spatial cognition discrimination. This conspicuous spatial configuration disability appeared as early as 1 week after L-BMAA treatment, which preceded the emergence of the observed motor functional impairments. However, the task also showed that treatment with C16+Ang-1 could reverse this memory disorder, while the L-serine treatment and the combined treatment produced even more remarkable effects (Table 3).

**Table 3:** The discrimination scores (%) of different groups showing that L-BMAA treatment impaired the object discovery ability at an early stage following injection, while treatment with C16+Ang-1 alleviated the memory disorder and the L-serine and combined treatments produced more remarkable effects.

	Normal	Vehicle	C+A	L-ser	Combined
0 day	68±4.3	63±4.34	66±3.12	67±3.21	68±2.9
1 week	70±2.11**^&^	43±3.27	54±5.21*	56±4.13*	54±2.19*
2 weeks	68±4.22**^&^	40±3.45	59±2.14*^&^	57±6.43*	56±3.29*
3 weeks	69±3.22**^&^	42±2.48	63±7.2**	62±3.58**^&^	59±2.46**
4 weeks	67±5.87**^&^	40±1.9	52±4.65*^&^	56±5.6*^&^	68±4.22**
5 weeks	68±7.23**^&^	41±3.56	55±3.79*^&^	58±2.98*^&^	63±2.35**
6 weeks	69±5.85**^&^	39±3.77	55±4.22*^&^	57±4.32*^&^	66±3.7**
7 weeks	70±3.49**^&^	42±4.98	54±5.4*^&^	55±3.45*^&^	64±4.39**
8 weeks	70±4.26**^&^	40±2.77	53±4.97*^&^	54±6.31*^&^	65±2.8**

C+A, C16+Ang-1; L-ser, L-serine; combined, C16+Ang-1 and L-serine. Data are expressed as mean±SD, n=10. *p<0.05 vs vehicle group, ^&^p<0.05 vs group receiving combined treatment.

### C16+Ang-1 with L-serine treatment attenuated the microglia activation, decreased the serum IL-6 level, reduced 3-NT and ROS/nitrogen species production induced by L-BMAA injection to a greater extent than L-serine treatment alone

**Figure 2:**
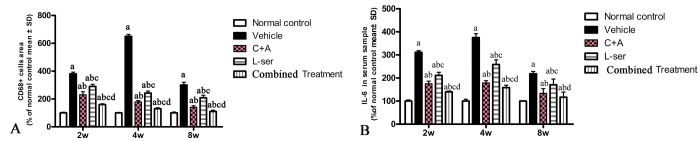
(**A**) CD68, a marker of activated microglia and extravasated macrophages, reached a maximal level at 4 weeks and then decreased considerably by 8 weeks after L-BMAA injection. Treatment with C16+Ang-1, L-serine, or both combined all obviously alleviated inflammatory cell infiltration, with the C16+Ang-1 and combined treatments producing better effects than treatment with L-serine alone. (**B**) Moreover, the serum IL-6 level increased after L-BMAA injection, and the changes in IL-6 and CD68 expression were similar. Administration of C16+Ang-1 and the combined treatment of C16+Ang-1 with L-serine also produced more remarkable inhibitory effects on IL-6 expression compared with L-serine treatment only. (a) p<0.05 vs normal rats; (b) p<0.05 vs vehicle-treated rats; (c) p<0.05 vs. C16+Ang-1–treated rats; (d) p< 0.05 vs. L-serine–treated rats.

CD68, a marker of activated microglia and extravasated macrophages, was increased by L-BMAA initially, reaching a maximal level at 4 weeks but declining appreciably by 8 weeks after injection (Fig. 2G,L). C16+Ang-1 treatment and the combined treatment markedly alleviated the infiltration of inflammatory cells, and the combined treatment produced better results than those observed with L-serine treatment alone (Fig. 2P). Moreover, the serum level of IL-6, which can induce a transcriptional inflammatory response, followed a trend similar to CD68 expression after L-BMAA injection, and treatment with C16+Ang-1 and L-serine plus C16+Ang-1 remarkably decreased IL-6 expression compared with L-serine treatment alone (Fig. 2Q).

**Figure 3:**
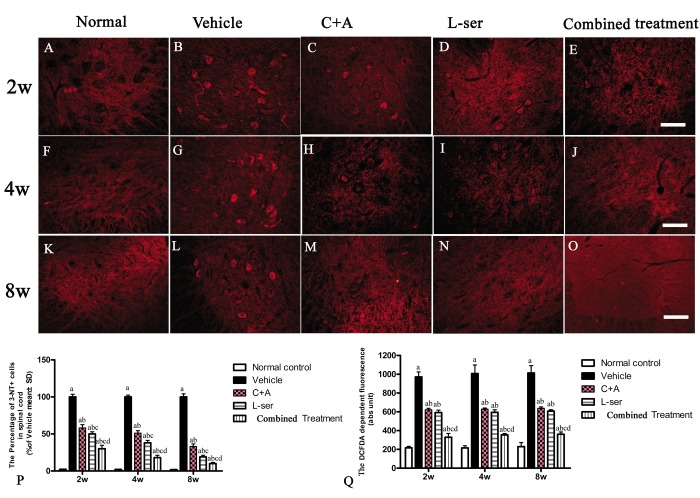
Immunostaining of 3-NT in lumbar spinal cord sections. The clear increase in 3-NT staining in spinal cord sections from the vehicle-treated rats from 2–8 weeks post-L-BMAA injection was clearly reduced by C16+Ang1 and L-serine treatment, and the combined treatment produced the most significant effects as revealed by cell quantification (**P**). (**Q**) ROS production. The amount of ROS produced in the vehicle-treated model group was significantly greater than the levels in the other groups. C16+Ang-1 and L-serine treatments, individually, reduced the ROS level, and the combined treatment led to the lowest ROS level among all the treated groups. (a) p<0.05 vs normal rats; **(**b) p<0.05 vs vehicle-treated rats; (c) p<0.05 vs. C16+Ang1–treated rats; (d) p<0.05 vs. L-serine–treated rats. Scale bar = 100 μm.

Immunostaining for 3-NT, a special marker of nitrogen species production, in sections of lumbar spinal cords showed clearly enhanced labeling in the vehicle-treated rats from 2–8 weeks after L-BMAA injection, which was markedly attenuated by C16+Ang1 treatment and L-serine treatment (Fig. 3). The combined treatment yielded the most significant effects as revealed by cell quantification (Fig. 3P). The production of ROS in the different groups is shown in Fig. 3Q. The amount of ROS produced in vehicle-treated model group was significantly higher than that in the other groups. Notably, while the ROS level was diminished in the C16+Ang-1 and L-serine treated groups, the group that received combined treatment exhibited the lowest ROS levels starting from the early stage (2 weeks after L-BMAA injection) to the late stage (8 weeks after L-BMAA injection; Fig. 3Q).

### L-BMAA augmented GSK3β and reactive gliosis, enhanced intracellular movement of cytosolic TDP-43 protein, and decreased GLT-1 expression, and these effects were effectively reversed by C16+Ang-1 and further reversed by L-serine treatment and the combined treatment

**Figure 4:**
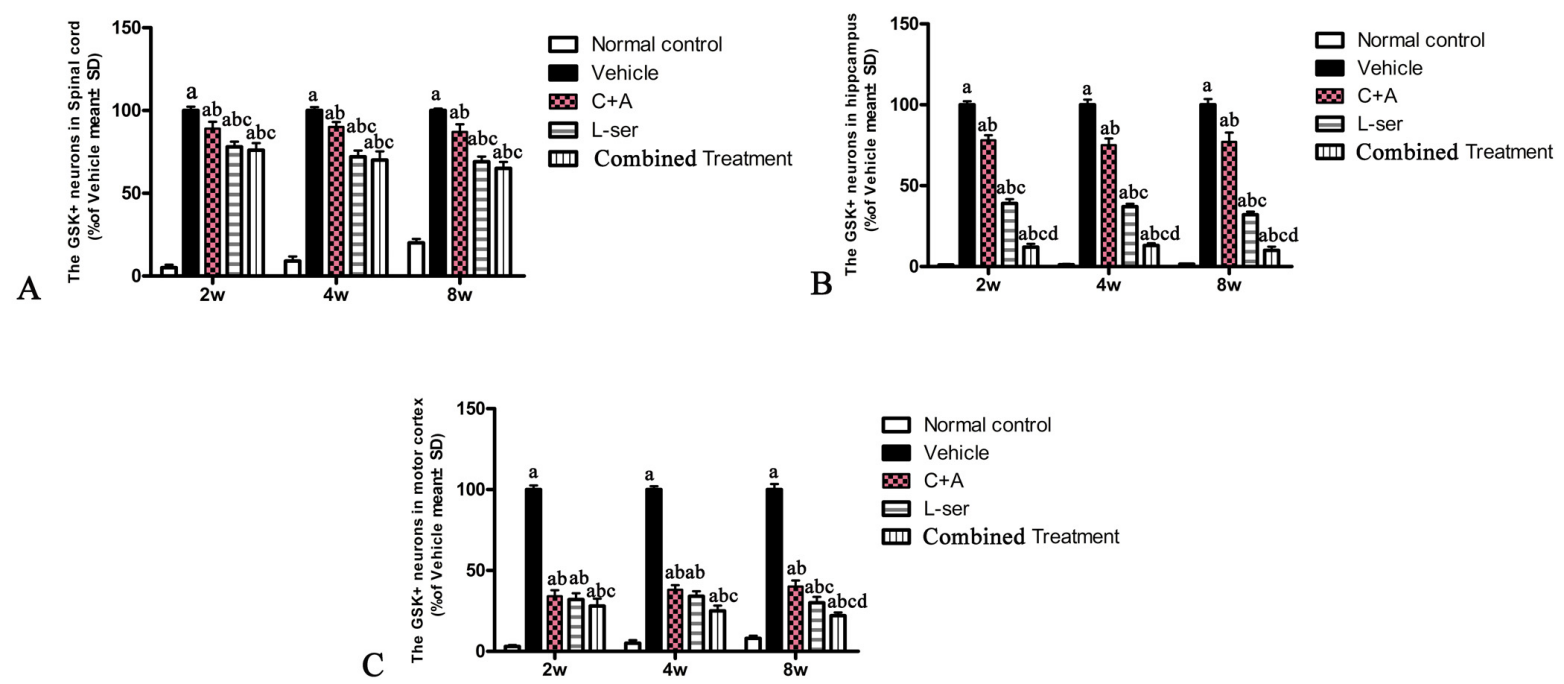
Increased expression of GSK3β in the spinal cord (**A**), hippocampus (**B**) and motor cortex (**C**) was observed in vehicle-treated model rats. The number of GSK3β-positive neurons was remarkably decreased by C16+Ang1 treatment and reduced to an even greater degree after L-serine treatment. The combined treatment group showed the lowest GSK3β cellular expression according to the results of cell quantification. (a) p<0.05 vs normal rats; (b) p<0.05 vs vehicle-treated rats; **(c**) p<0.05 vs. C16+Ang1–treated rats; (d) p<0.05 vs. L-serine–treated rats.

**Figure 5:**
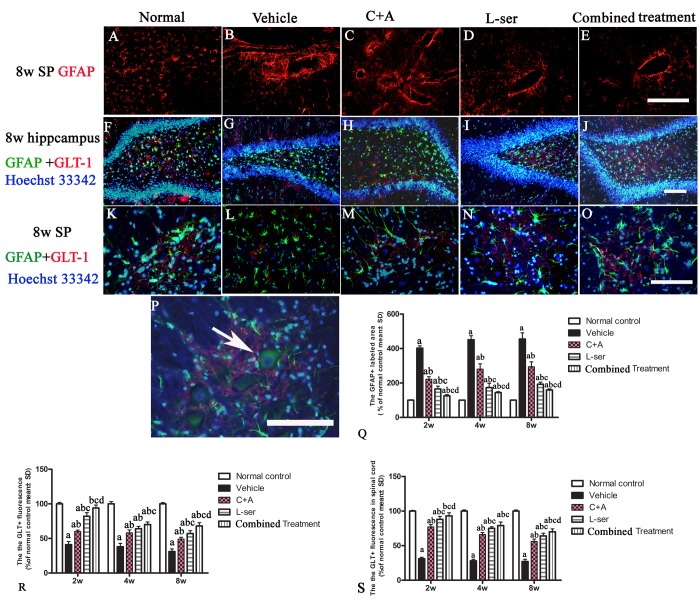
(**A**-**E**) Vehicle-treated model rats exhibited pronounced reactive gliosis as revealed by TRITC-conjugated GFAP immunofluorescence staining. SP: spinal cord. (**A**) Normal astrocytes in the spinal cord. (**B**) Glial scarring following L-BMAA injection in the vehicle-treated model group. (**C**-**E**) C16+Ang-1treatment alleviated reactive astrogliosis. (**Q**) L-serine treatment resulted in fewer abnormally proliferating astrocytes, and the combined treatment produced more pronounced effects according to cell quantification. (**F**, **K**, **P**) In normal control rats, intense GLT-1 labeling appeared in astrocytes around neurons of the DG region of the hippocampus and ventral horn motor neurons (arrow in P), in contrast to the pronounced decrease in GLT-1 labeling around ventral horn motor neurons in the vehicle-treated model rats. Treatment with C16+Ang-1, L-serine, and especially combined treatment with both results significantly increased expression of GLT-1. Scale bar = 100 μm. (a) p<0.05 vs normal rats; (b) p<0.05 vs vehicle-treated rats; (c) p<0.05 vs.C16+Ang-1–treated rats; (d) p<0.05 vs. L-serine–treated rats.

In the vehicle-treated model group, L-BMAA injection led to augmented expression of GSK3β in the spinal cord, hippocampus and motor cortex (Fig. 4). Staining for GFAP, a marker of astrocytes, showed the increased proliferative activity of these cells and formation of a visible glial scar. However, astrocytic glutamate transporter (GLT-1), another marker of astrocyte pathology, was obviously decreased in the vehicle-treated group, implying a reduction in the astrocytic glutamate transport capacity (Fig. 5). The up-regulation of GSK3β, reactive gliosis and down-regulation of GLT-1 were all significantly reversed by C16+Ang-1 treatment, and further still by L-serine treatment. The combined treatment group showed the best effects (Figs. 4-5).

**Figure 6:**
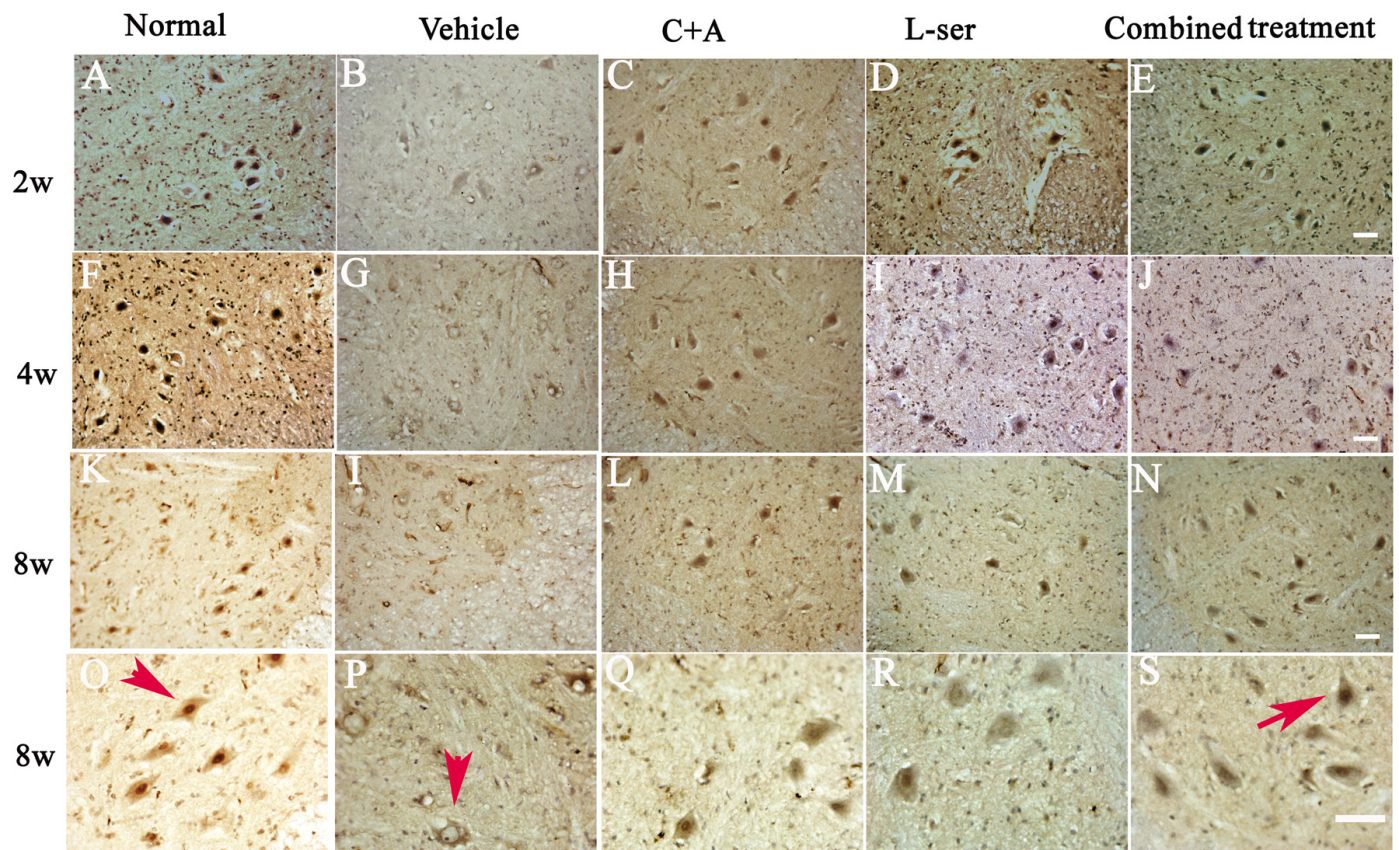
L-BMAA injections stimulated the emergence of cytosolic TDP-43 immunoreactive aggregates in spinal cord motor neurons. Scale bar = 100 μm. In the motor neurons of normal control rats, TDP-43 expression was primarily confined to the nucleus (indicated by the arrow in **O**). However, approximately 6 weeks after L-BMAA injection, a decline in the distinct nuclear expression of TDP-43 was accompanied by the emergence of cytosolic staining in a proportion of degenerating motor neurons. By 8 weeks after L-BMAA injection, a pronounced decline in TDP-43 expression in the nuclei of damaged motor neurons was observed (indicated by the arrow in **P**), and the nuclei showed a bulging and vacuolated morphology. With C16+Ang-1 or L-serine treatment, the TDP-43 labeling in large motor neurons was mainly localized in the nuclei compared with that in the control group, and this phenomenon was more visible in the combined treated group (shown by the arrow in **S**).

TDP-43 expression is usually confined to the nucleus, as displayed in Figure 6 in the motor neurons in the normal control (Fig. 6, arrow in O). In the vehicle-treated group, reduced nuclear expression of TDP-43 was accompanied by the emergence of some cytosolic staining in some deteriorating motor neurons 2–4 weeks after L-BMAA injection (Fig. 6B, G). Decreased TDP-43 expression in the nuclei of injured motor neurons was noted 8 weeks after L-BMAA administration, and the nuclei displayed a bulge and vacuolated morphology (indicated by arrows in Fig. 6P). However, in the C16+Ang-1 or L-serine–treated rats, TDP-43 labeling in large motor neurons mainly accumulated in the nucleus unlike in the control group, and this effect was even more obvious in the combined treatment group (indicated by arrow in Fig. 6S).

### L-BMAA injection increased APP and hyper-phosphorylated tau expression, and C16+Ang-1 and L-serine treatments inhibited these phenomena, with the combined treatment producing the best effects

**Figure 7:**
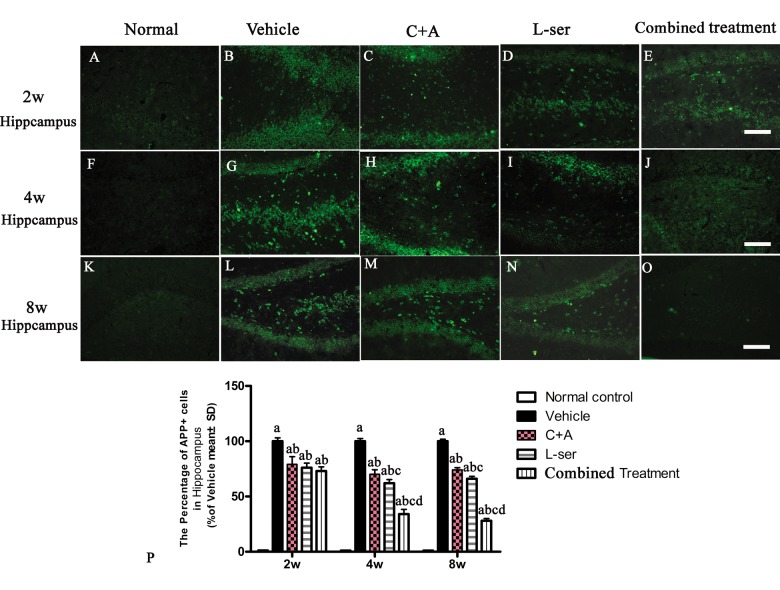
Immunostaining for APP in sections of the hippocampus. The clear increase in APP staining in the vehicle-treated model rats from 2–8 weeks after L-BMAA injection was obviously reduced by C16+Ang-1 and L-serine treatment individually, and the combined treatment with both led to the most significant effect as revealed by cell quantification. (a) P<0.05 vs normal rats; (b) p<0.05 vs vehicle-treated rats; (c) p<0.05 vs. C16+Ang-1–treated rats;(d) p<0.05 vs. L-serine–treated rats. Scale bar = 100 μm.

**Figure 8:**
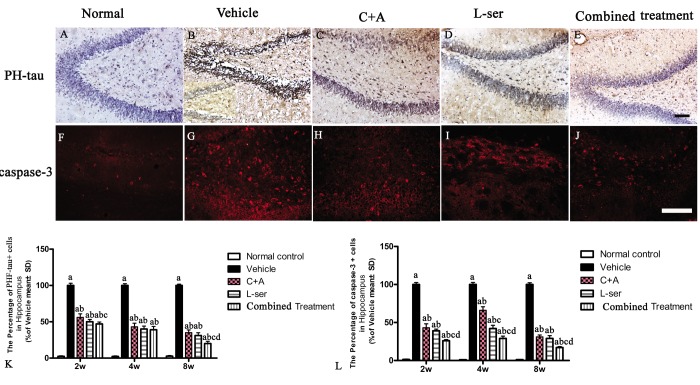
(**A**-**E**) Microscopic pathology in L-BMAA–injected vehicle-treated group. Immunostaining showed that hyperphosphorylated (PHF) tau expression was increased in the neurons of the hippocampus and spinal cord anterior horn compared with that in the normal control rats. (**B**) Enlarged image was inserted to show the positive stained neurons in the vehicle-treated group. (**F**-**J**) Caspase-3 expression was increased in the hippocampus after L-BMAA administration as revealed by immunofluorescence staining. (**K**) C16+Ang-1 and L-serine treatments inhibited the elevated expression of PHF-tau, and the combined treatment showed the most obvious effects. (**L**) Treatment with C16+Ang-1, L-serine, and especially both obviously reduced the number of caspase-3–positive neurons labeled with red fluorescence. Scale bar = 100 μm (a) p<0.05 vs normal rats; (b) p<0.05 vs vehicle-treated rats; (c) p<0.05 vs. C16+Ang-1–treated rats; (d) p<0.05 vs. L-serine–treated rats.

Increased labeling of APP, the main mediator of proamyloidogenic pathway activation, was detected in the hippocampus of the vehicle-treated model rats (Fig. 7), and immunostaining for hyper-phosphorylated tau (abnormal phosphorylation of tau) was increased in neurons of the hippocampus compared with that in the normal control rats (Fig. 8B). Both phenomena were obviously attenuated by C16+Ang-1 and L-serine treatment, and the group that received combined treatment showed the most significant effects as revealed by cell quantification (Figs. 7 and Fig. 8K).

### L-BMAA induced neurofilament disintegration and axonal destruction, which were reversed by C16+Ang-1 and L-serine and to an even greater extent by the combined treatment

**Figure 9:**
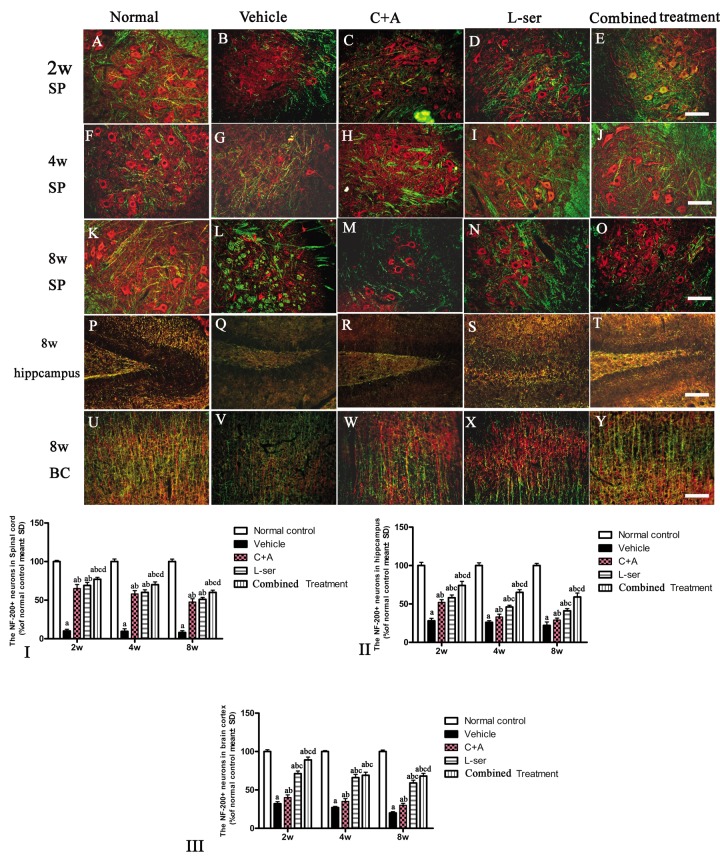
After the rats had received injections of 300 mg/kg BMAA each day for 3 days, morphological changes were observed in the motor neurons of the lumbar ventral horn (**A**-**O**), hippocampus (**P**-**T**), and brain motor cortex (**U**-**Y**) at 2w, 4w and 8w post-injection. NF-200 (red, a specific marker for neurofilament in neurons) and MBP (green, a specific marker for myelin) double immunolabeling, which recognizes neurofilament proteins pivotal for maintaining large neurons with extensively myelinated processes, allowed visualization of changes in somatic and dendritic morphology and revealed structural injury. Scale bar = 100 μm. In the vehicle-treated model group, extensive disintegration of dendritic processes and a lack of NF-200 staining both in somatic and dendrites of neurons as well as atrophy of lumbar ventral horn motor neurons were noted (**L**); a drastic decrease in NF-200 staining was also discernible at 2 months after L-BMAA injection in the hippocampus (**Q**) and motor cortex (**V**). However, C16+Ang-1 and L-serine treatments could attenuate the loss of axons in the CNS after L-BMAA administration, and the combined treatment produced the most pronounced effects. Transverse sections of the anterior horn of the lumbar spine are denoted with SP, and coronal sections of the motor cortex are denoted with BC. (I-III) Quantification of relative fluorescence of NF200-positive cells. (a) p<0.05 vs normal rats; (b) p<0.05 vs vehicle-treated rats; (c) p<0.05 vs. C16+Ang-1–treated rats; (d) p<0.05 vs. L-serine–treated rats.

Loss of dendrites in neurons as well as neurofilament proteins pivotal in maintaining large neurons with extensively myelinated processes was revealed by double-staining for NF-200 and MBP in the spinal cord anterior horn, hippocampus and motor cortex of vehicle-treated model rats (Fig. 9G,L,Q,V). Visibly swollen motor neurons, atrophy and significant loss of NF-200 staining also were observed (Fig. 9L,Q,V). However, C16+Ang-1 and L-serine treatments attenuated the axonal destruction in the CNS after L-BMAA administration, and the combined treatment produced more pronounced effects as shown by quantification of NF-200+ cells (Fig. 9).

**Figure 10:**
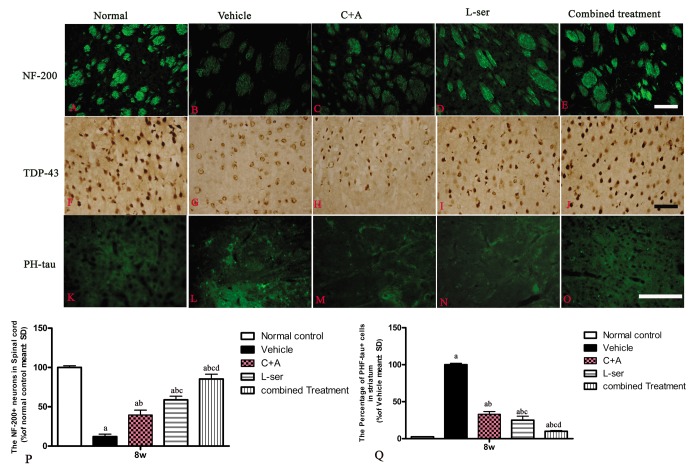
At 8 weeks post-injection, neurofilament disintegration and axonal destruction were found in basal ganglia of vehicle-treated group (B) in contrast with the normal control. At the same time point, intracellular movement of cytosolic TDP-43 protein (**G**) and high expression of hyper-phosphorylated tau (**L**) were detected in the striatum of the vehicle-treated group, while the C+A (**C**, **H**, **M**) and L-ser treatment (**D**, **I**, **N**) both were found to alleviate these effects in some extent, with the combined treatment (**E**, **J**, **O**) producing the best effects. (**P**, **Q**) Quantification of relative fluorescence of NF-200 (**P**) and PHF-tau (**Q**) staining in cells. (a) p<0.05 vs normal rats; (b) p<0.05 vs vehicle-treated rats; (c) p<0.05 vs. C16+Ang-1–treated rats; (d) p<0.05 vs. L-serine–treated rats.

At 8 weeks post-injection, intracellular movement of cytosolic TDP-43 protein and high expression of hyper-phosphorylated tau were detected in the striatum. Furthermore, neurofilament disintegration and axonal destruction also appeared in the striatum as shown by NF-200 immunostaining. All of these signs suggested chronic damage of the basal ganglia (Fig.10).

### L-BMAA induced mitochondrial deterioration, autophagy, and neuronal apoptosis, and these phenomena were reversed by C16+Ang1 treatment, further alleviated by L-serine treatment, and most significantly attenuated by the combined treatment

**Figure 11:**
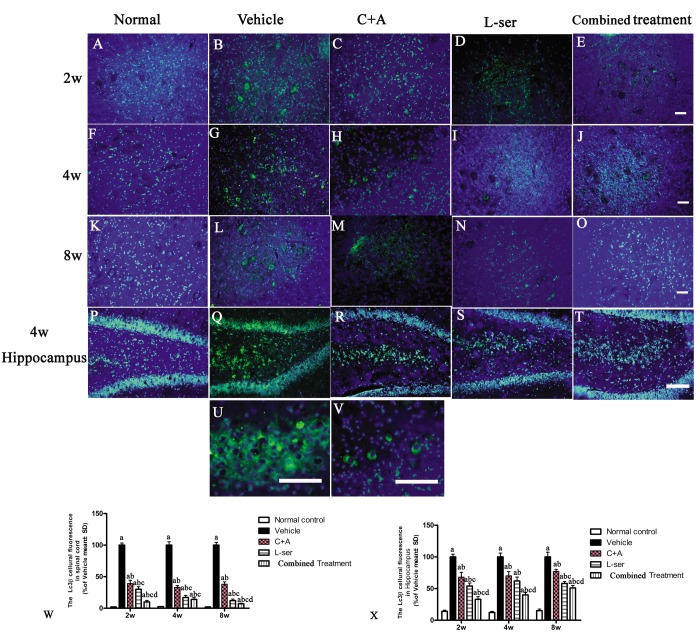
Representative fluorescence microscopy images of the spinal cord anterior horn and hippocampus. LC3B fluorescence appears green and Hoechst 33342 (nucleus stain) appears blue. (**A**-**T**) Treatment with C16+Ang1 and L-serine, separately, reversed the up-regulation of LC3 expression compared with vehicle-treated group after L-BMAA injection at different time points, and the combined treatment showed a stronger effect on LC3 expression than either individual treatment group. (**U**-**V**) Enlarged images of sections from the hippocampus of the vehicle-treated model group (**Q**) and from the spinal cord of the C16+Ang-1–treated group (**H**) show the cytosolic localization of LC3 staining. (**W**, **X**) Quantification of relative fluorescence of LC3-positive cells. (a) p<0.05 vs normal rats; (b) p<0.05 vs vehicle-treated rats; (c) p<0.05 vs. C16+Ang-1–treated rats; (d) p<0.05 vs. L-serine–treated rats.

In most neurodegenerative conditions, extensive neuronal destruction and autophagy occur. Immunohistochemical staining was employed to detect the presence of the autophagy marker LC3B. L-BMAA injection decreased LC3B expression, and LC3B fluorescent labeling was observed in the cytoplasm of both spinal cord anterior horn neurons and hippocampal neurons (Fig.11).

**Figure 12:**
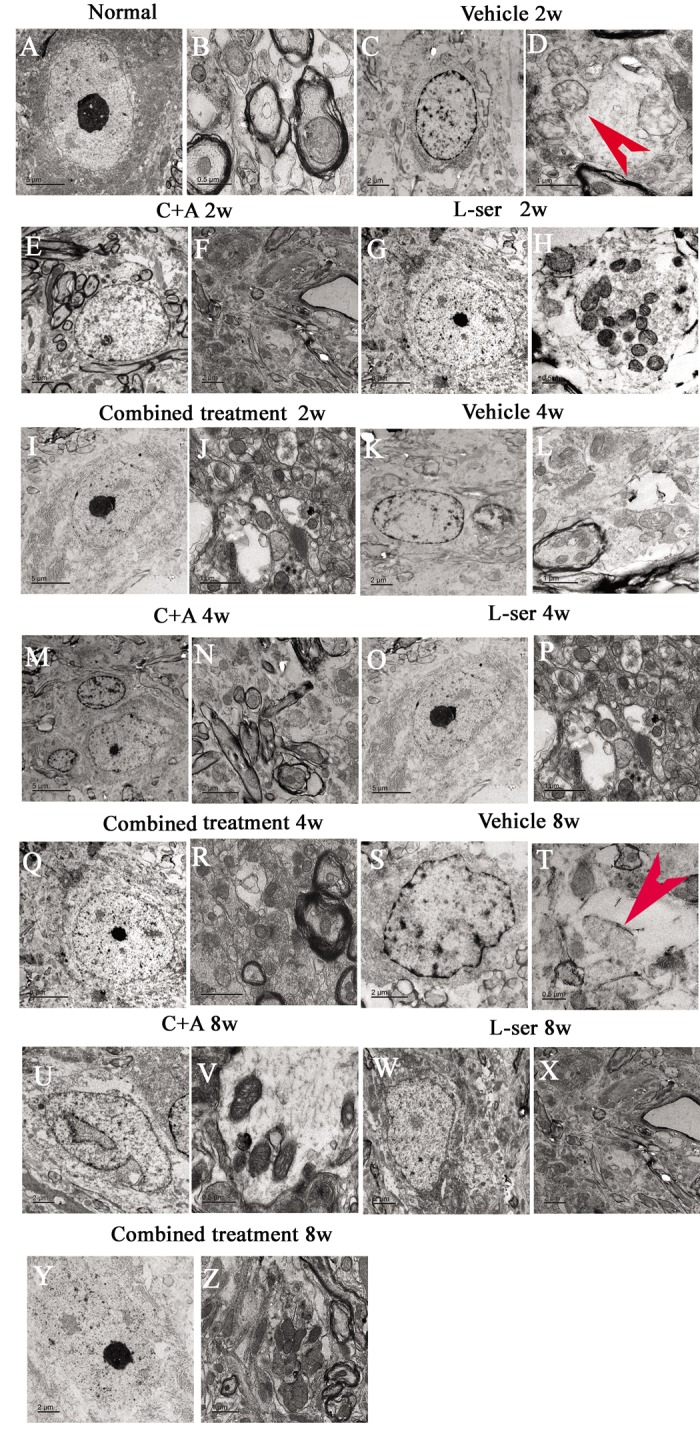
Representative electron micrographs showing mitochondrial atrophy, fragmented vacuoles, loose and fused alterations of the myelin sheath, and apoptotic features of the nuclei of spinal cord anterior horn motor neurons in the vehicle-treated model rats, as well as the attenuating effects of treatment with C16+Ang-1, L-serine, or both. (**A**–**B**) Rats in the control group showed (**A**) normal neuronal nuclei with uncondensed chromatin and (**B**) normal myelinated axons with dark, ring-shaped myelin sheaths surrounding the axons, with normal shaped mitochondria. (**C**-**D**, **K**-**L**, **S**-**T**) In vehicle-treated model rats 2–8 weeks after L-BMAA injection, fragmented vacuoles and loose and fused alterations on the myelin sheath were found, as well as mitochondrial malformations such as vacuolization and swollen cristae (arrows in **D**). At 8 weeks after L-BMAA injection (**T**), some mitochondria had a completely atrophied appearance, and their cristae had vanished. More drastic alterations in demyelination and axonal loss were found (**L**, **T**), and neurons also showed apoptotic features with a contracted nucleus and condensed, fragmented, and marginated nuclear chromatin (**C**, **K**, **S**). By contrast, in rats treated with C16+Ang-1(**E**-**F**, **M**-**N**, **U**-**V**), L-serine (**G**-**H**, **O**-**P**, **W**-**X**), and both (I-J, Q-R, Y-Z), newly formed myelin sheaths were observed surrounding the intact axons, and the morphology of mitochondria and nuclei were relatively normal. This was particularly obvious in the group that received the combined treatment (**Y**, **Z**). (**B**, **H**, **T**, **V**) Scale bar = 500 nm; (**D**, **J**, **L**, **P**, **R**, **Z**) scale bar = 1 μm; (**C**, **E**, **F**, **K**, **N**, **S**, **U**, **W**, **X**, **Y**) scale bar = 2 μm; (**A**, **G**, **I**, **M**, **O**, **Q**) scale bar = 5 μm.

**Figure 13:**
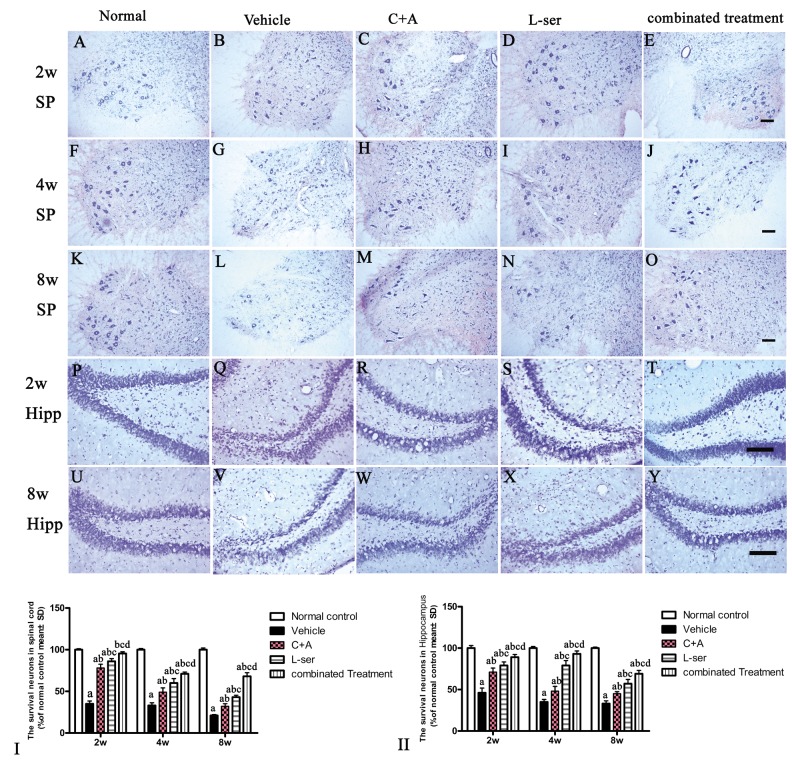
The gradual loss of neurons in the CNS of L-BMAA–treated rats was revealed by Nissl staining. However, the number of surviving neurons was markedly increased by C16+Ang-1 treatment and further still by L-serine treatment. The combined treatment produced the best effects as revealed by cell quantification.SC: spinal cord; H: hippocampi. Scale bar = 100 µm. Neurons were counted in three visual fields per section taken at 200× magnification under bright field viewing. (I-II): The rate of surviving neurons (shown as % of normal control) was calculated based on the neuron number. (a) p<0.05 vs normal rats; (b) p<0.05 vs vehicle-treated rats; (c) p<0.05 vs. C16+Ang-1–treated rats; (d) p<0.05 vs. L-serine–treated rats.

EM examination revealed alterations including mitochondrial vacuolization and distended mitochondrial cristae after L-BMAA injection (arrows in Fig. 12D), and the myelin sheath of nerve fibers exhibited cleaving, vacuolization, and loose and fused changes (Fig. 12L, T). At the late phase (8 weeks), some of the mitochondria had undergone complete deterioration with total loss of cristae (arrows in Fig. 12T). Neurons exhibited features of apoptosis with a contracted nucleus and compressed, disintegrated and marginated nuclear chromatin (Fig. 12C,K,S). L-BMAA activated the pro-apoptotic enzyme caspase-3 as evidenced by immunofluorescence staining (Fig. 8L). Nissl staining revealed progressive neuronal destruction in the spinal anterior horn and hippocampi of the model rats (Fig. 13). The C16+Ang-1 and L-serine treatments attenuated the elevated expression of LC3, reduced the number of caspase-3–positive neurons, and maintained essentially normal morphological features of the nuclei and mitochondria. Moreover, the combined treatment yielded improved results compared with the single treatments (Figs. 8,11,12,13).

## DISCUSSION

Environmental causes of ALS/PDC are responsible for the onset of sporadic cases. L-BMAA, an amino acid not derived from proteins, was first implicated with the heightened occurrence of ALS/PDC on the small island of Guam and was considered as a potential environmental factor in neurodegenerative diseases including ALS and Alzheimer’s disease [14]. L-BMAA exerts toxic actions toward motor neurons, including a direct agonist action toward NMDA and AMPA receptors, generation of oxidative stress, and down-regulation of glutathione [15]. It is very likely that L-BMAA can induce an abnormal folding of proteins in neurons, which is the hallmark of neurodegeneration [16]. Although the connection between L-BMAA and sporadic ALS requires corroboration using a primate model of L-BMAA-induced progressive neurodegeneration, the recently established rodent animal models have been extensively applied in investigations aimed at elucidating the mechanisms of ALS/PDC [17-19].

Translation is a process by which amino acids are incorporated into proteins with great precision [16]. Although errors rarely occur in translation, when errors do occur they could bring about the mischarging of tRNA by an inappropriate amino acid, leading to the formation of misfolded or truncated proteins, eventually resulting in cellular damage and neurodegeneration [16,20]. The action of L-BMAA at various levels in cells may increase the risk of neurodegenerative diseases through effects such as gliotoxicity and neurotoxicity and synergistic adverse effects in conjunction with other cyanotoxins [21]. Even at low doses, L-BMAA treatment resulted in neurobehavioral disturbances during both the postnatal period and adulthood in rodent models [22-24]. Because L-BMAA is incorporated into proteins in lieu of L-serine by mistake to bring about protein misfolding and aggregation, this erroneous incorporation may be reversed by treatment with L-serine [16].

Previous studies showed that L-BMAA can induce peptide changes in newborn mice and cognitive deficiencies in adult mice [25]. Rats treated with L-BMAA demonstrated pathological changes in the motor neurons [26]. Electron microscopic examination of the spinal cords of these rats uncovered a variety of modifications consistent with the incorporation of a nonprotein amino acid into proteins, including increased caspase-3 expression, apoptosis, distended cristae and disorganization of mitochondria [16]. All of the pathophysiological changes demonstrated in previously reported investigations were in line with the results of the present investigation. The up-regulation of 3-NT, APP, PHF-tau, GFAP, GSK3, and LC3 along with the down- regulation of GLT-1 and NF-200 following L-BMAA injection were also revealed in our investigation. These changes indicate the successful establishment of an ALS/PDC model using consecutive intravenous L-BMAA injections.

Like many other neurodegenerative diseases, sporadic ALS is characterized by neuroinflammation, including infiltration of lymphocytes and macrophages, activation of microglia and reactive astrocytes, as well as the involvement of the complement system [27]. Neuro-inflammation is recognized as a key contributor to motor neuron degeneration and disease progression [28]. The inflammatory environment in ALS changes with disease progression. Resident glial cells and infiltrating immune cells are considered among the major producers of ROS and reactive nitrogen species, which contribute to the pathological conditions of the CNS, including phagocytosis and apoptosis of the spinal cord motor neurons and neurons in the dentate gyrus (DG) region of the hippocampus [6,8,29]. The Alzheimer’s disease-like pathology is synergistic and dependent on oxidative stress and inflammation; thus, administration of anti-inflammatory agents might alleviate the disease outcome [30]. However, we noticed that the anti-inflammatory effect of L-serine was much less than that observed with C16+Ang-1 treatment in the present study.

In our previous studies in an experimental autoimmune encephalomyelitis (EAE) model, Tie2 and ανβ3 integrin, which are the targets of Ang-1 and C16, respectively, were increased in the vascular endothelial cells (ECs) of blood vessels [31]. Furthermore, in addition to competitively inhibiting αvβ3 integrin signaling and blocking binding of inflammatory cells to the endothelium to eventually prevent leukocyte transmigration, C16 can also function as a ανβ3 agonist to promote therapeutic angiogenesis by endothelial cells [32]. Despite the effects of inhibiting the expression of adhesion factors and thwarting the adhesion of inflammatory cells, the major actions of Ang-1 are ascribed to the preservation of the integrity of ECs, enhanced survival of ECs, and reduced blood vessel leakage through the Ang-1–Tie2 system [9]. Although the mechanisms of the activities of Ang-1 and C16 partly overlap, the synergistic effects of the two drugs based on their targeting of different specific receptors lead to alleviation of inflammation, reduction of axonal loss and mitigation of electrophysiological dysfunction [10,11]. Because the inhibition of inflammatory cell transmigration mechanisms by C16+Ang-1 treatment is nonspecific, the micro-environment within the CNS could be improved, thus reducing the production of reactive oxygen and nitrogen species and finally alleviating CNS neuroinflammation caused by different diseases and different mechanisms.

In reports on the toxic effects of L-BMAA, long-term alterations including inhibited expression of proteins implicated in energy metabolism and intracellular signaling in the adult hippocampus were found after administration of a dose of 150 mg/kg, and a higher dose of L-BMAA (460 mg/kg) caused serious damage in the adult hippocampus, including neuronal degeneration, cell loss, calcium deposits, and astrogliosis [21]. In the present study, with a L-BMAA dose of 300 mg/kg, obvious impairment in object–place recognition ability was detected in the vehicle-treated model rat group 1 week after L-BMAA injection, which was even earlier than the onset of visible motor functional disabilities as well as pathologic changes in MEP and MUPs on electromyography. It was recently reported that knocking out TDP-43 in the hippocampus and cortex elicits severe neuronal loss due to depletion of TDP-43 [33], and accumulation of TDP-43 in the cytoplasm might contribute to the regulation of the subcellular localization of APP and tau aggregation [34]. In the present study, cytosolic aggregates of TDP-43 and accumulation of the microtubule-associated protein tau and APP within degenerating neurons of the hippocampus accompanied by multiple functional impairments, all of which represent the characteristics of ALS/PDC, were observed after L-BMAA treatment and then alleviated by combined treatment with C16+Ang-1 and L-serine.

Previous studies have shown that acute perfusion of high doses of BMAA through the striatum of the rat via a microdialysis cannula does not cause a clear damage to the dopaminergic terminals of the nigrostriatal system [35], Murch et al. [36] suggested that the bound form of BMAA within the brain tissues may function as an endogenous neurotoxic reservoir, accumulating and being transported between trophic levels before its subsequent release during digestion and protein metabolism. This endogenous neurotoxic reservoir in the striatum can slowly release free BMAA, thereby causing neurological damage over year or even decades, which may explain the observed long latency period for neurological diseases among the Chamorro people [36]. Moreover, previous studies demonstrated that in ALS with cognitive impairment (ALSci), widespread astrocytic tau deposition and the absence of nuclear TDP-43 immunoreactivity were found in the superior frontal cortex, anterior cingulate gyrus, entorhinal cortex, amygdala and basal ganglia [37]. In our study, we observed intracellular movement of cytosolic TDP-43 protein and high expression of hyper-phosphorylated tau in the striatum at 8 weeks post-injection. Furthermore, NF-200 immunostaining showed neurofilament disintegration and axonal destruction in the striatum, suggestive of chronic damage to the basal ganglia.

In conclusion, the results of the present study confirmed the benefit of L-serine treatment in the ALS/PDC rat model induced by L-BMAA injection. L-serine application yielded better effects than C16+Ang-1 treatment alone for alleviating apoptotic and autophagic changes, reducing the disorganization of mitochondria, reactive gliosis and axonal loss in the spinal cord and hippocampus, rectifying the abnormal changes of protein expression, and improving cognition and electrophysiological dysfunction, but not for improving the inflammatory micro-environment in the CNS. Therefore, C16+Ang-1 could be beneficial as a supplement for promoting the effects of L-serine treatment through its targeting of different molecules, and the best effects were produced in the group receiving combined treatment in the present study, with no evidence of adverse side effects of the combined treatment. Efforts to adjust the dosages of the different components of this combined therapy are ongoing in our laboratory.

## MATERIALS AND METHODS

### Animals and allocation to different experimental groups

One hundred forty-three Adult Sprague-Dawley rats (male, 150–200 g) were obtained from the Laboratory Animal Center of Zhejiang University and allocated to a normal control group (n=11) or one of the following ALS/PDC model treatment groups: (1) vehicle control (n=33), (2) L-serine treatment (n=33), (3) C16+Ang-1 treatment (n=33), and (4) L-serine and C16+Ang-1 co-treatment (n=33). In each treatment group, 11 rats were killed at the time points 2, 4, and 8 weeks after intraperitoneal (ip) L-BMAA injection. From six of these rats, tissues were collected for histological evaluation, immunohistological and immunofluorescence staining, and transmission electron microscopic observation, while tissues from the remaining five rats were used for enzyme-linked immunosorbent assay (ELISA), western blotting analysis, and reactive oxygen species (ROS) analysis. Experiments involving animals were carried out following the regulations of the National Institutes of Health Guide for the Care and Use of Laboratory Animals.

### ALS/PDC induction and drug treatment

A solution of L-BMAA from Sigma-Aldrich (St. Louis, MO, USA) in sterile distilled water (150 mg/ml) was prepared. In the model rats which weighed approximately 250 g, ALS/PDC was induced, by injection of 75 mg L-BMAA (300 mg/kg) into the caudal vein daily for a total of 3 consecutive days [17]. The normal control rats received injections of 0.5 ml distilled water instead.

One milliliter of L-serine solution (62.5mg/mL in distilled water) was injected intraperitoneally daily for 1 week into the model rats in the groups treated that received L-serine treatment or treatment with both L-serine and C16+Ang-1. The normal control rats received intraperitoneal injections of distilled water (1ml) instead.

The C16 peptide (KAFDITYVRLKF) was synthesized as described previously [10,11], and then the C16 peptide solution (4 mg/mL) was prepared in phosphate-buffered saline (PBS). A solution of Ang1 peptide (Shanghai Science Peptide Biological Technology Co., Ltd., China) in distilled water (800 µg/mL) also was prepared [12]. For the model rats that received Ang1+C16 treatment or L-serine plus Ang1+C16 treatment, 0.5ml Ang1 solution (400 µg) and 0.5ml C16 solution (2 mg) were injected intravenously through the caudal vein daily for 1 week. The first injection was administered immediately after induction of ALS/PDC. Rats in the normal control and vehicle-treated model group received intravenous injections of PBS.

### Tests of motor function

Motor function scales were previously established for assessing neurological dysfunction in ALS/PDC [38]. These assessments in the present study were carried out by investigators blinded to the various groups of the experimental animals.

### Rotarod test

The rotarod test records the duration that the rat can stay on a spinning rod before falling off. For this test, rats were positioned on the rotarod (radius, 4.5 cm and width, 8 cm; KN-75, Natsume Seisakusho Co., Ltd.), which was then rotated at 15 rpm in the forward direction. Rats were drilled two or three times weekly before testing, and animals that could stay on the cylindrical rod for more than 2 min were selected for the experiment. This test was performed weekly for 8 weeks.

### Inclined plane test

In the test, each rat was positioned on a rubber-coated board that could be inclined at different angles, and the greatest angle at which the rat could stay in position for 5 s was recorded. The position of the board was adjusted at 10° intervals, from an angle of 10° to an angle of 90°. The test was performed weekly for 8 weeks.

### Forepaw gripping ability

Before testing of forepaw grip strength, rats were handled for 5 min and exposed to a grip strength meter (GSM, TSE-Systems) for a couple of days. For testing, each rat was held by the tail just above the GSM bar. Once the rat reached the bar and gripped it with an unrestrained paw, it was then pulled away from the GSM bar in one smooth movement until it released its grip on the bar. The GSM estimated the force (g) of the grip at the moment immediately prior to the release of the bar [17].

### Object–place recognition task

The object–place recognition task was carried out as described previously [39]. Each behavioral run consisted of familiarization (F) and novel location (NL) with rest sessions prior to and following every session. The paradigm was conducted several times for each rat. Novelty preference was defined as the difference in the length of time each rat spent close to the object in the novel location, and the discrimination score was calculated as (NL–F)/(NL+F) in seconds [39].

### Electrophysiology examination

After injection of L-BMAA into the model rats, motor-evoked potential (MEP) and indexed motor unit potentials (MUPs) were recorded weekly. The stimulus was administered using an acupuncture pin electrode. The latency and amplitude of MEP were calculated as previously described [17]. Electronic signals were analyzed computationally.

MUPs were recorded by conventional electromyography using a disposable monopolar needle electrode (Dantec 13R1, recording surface area of 0.07 mm^2^). Contamination of the index motor unit by other motor units was minimized via the use of a window discriminator as previously described [17,40]. MUPs were deemed anomalous if the value exceeded one-fifth of the values in normal controls.

### Perfusion and tissue processing

As described above, rats in the treatment groups were killed 2, 4, and 8 weeks (n=6 per group at each time point) after L-BMAA injection. Each rat was subjected to anesthesia with sodium pentobarbital before the heart was perfused with cold saline followed by 4% paraformaldehyde in 0.1 M PBS (pH 7.4). The entire brain and spinal cord were excised and then half of the brain and 1 cm of the lumbar spinal cord were fixed in the same fixative for 4 h, before immersion in a 30% sucrose solution in PBS until the samples no longer floated. A Leica cryostat was used to prepare 20-µm coronal sections of the brain and 20-µm transverse sections of the spinal cord, which were then mounted on glass slides coated with 0.02% poly-L-lysine for later histological, immunohistological, and immunofluorescence staining analyses. The other half of each brain and the remaining spinal cord were fixed in 10% glutaraldehyde solution for imaging by transmission electron microscopy [17].

### Histological evaluation

Cresyl violet (Nissl) staining was employed to evaluate inflammation and the number of viable neurons within the sections of CNS tissues. Five sections of the cortex, hippocampi, and spinal anterior horn from each animal were chosen randomly, and micrographs of three fields of vision in each section were taken at 200× magnification. In each image, the numbers of neurons with distinct nucleoli as well as cell bodies with abundant endoplasmic reticulum were counted.

### Immunohistochemical staining

In preparation for immunohistochemical staining, slides of CNS tissues were warmed on a slide warmer for 20 min, and then a PAP pen (Invitrogen, Carlsbad, CA) was used to place a circle of wax around the sections. The slides were washed in 0.01 M Tris-buffered saline (TBS) for 10 min before tissue permeabilization in 0.3% Triton X-100/10% normal goat serum in 0.01 M PBS for 60 min. Finally, the slides were kept overnight at 4°C in solutions of primary antibodies, including monoclonal mouse antibodies anti-Tau-5 (1:500; Invitrogen), CD68/ED1 (1:100; Santa Cruz Biotechnology, Santa Cruz, CA), and anti-TAR DNA-binding protein 43 (TDP)-43 (1:1,000; Proteintech Group, Chicago, IL). After washing the next day, sections were exposed to the secondary antibody, biotinylated goat anti-rabbit/mouse IgG antibody (1:400; Vector Laboratories, CA) at room temperature for 1 h before development of staining with the avidin-biotin-peroxidase complex (ABC kit; Thermo Fisher Scientific, CA) via 5 minutes in DAB at a final concentration of 12mM in PBS with 0.003% H_2_O_2_). Finally, sections were counterstained with hematoxylin. Control sections were processed without incubation in the primary antibody to confirm specificity of the immunohistochemical labeling.

### Immunofluorescence staining

For immunofluorescence staining, sections were then exposed to the following primary antibodies for 15 h at 4°C: polyclonal rabbit anti-GSK3β (1:1,000; Thermo Fisher Scientific, Waltham, MA), anti-3-nitrotyrosine (3-NT, 1:300, Upstate Biotechnology, Waltham, MA), anti-LC3B (1:200, Novus Biologicals, Littleton, CO, USA), anti-GLT-1 (1:1,000; Chemicon, Temecula, CA), anti-GFAP (1:200; Thermo Fisher Scientific), anti-caspase-3 (1:500; Cayman Chemical, Ann Arbor, MI), anti-amyloid beta precursor protein (APP*,* 1:500; Abcam, Cambridge, MA), mouse anti-neurofilament 200 (NF-200, 1:500; United States Biological, Salem, MA), or myelin basic protein (MBP, 1:100; NE1018, Sigma-Aldrich). Sections were rinsed with PBS before incubation for 1 h at 37°C in a solution of TRITC (rhodamine)-conjugated goat anti-mouse or FITC-conjugated goat anti-rabbit IgG secondary antibodies (1:200; Invitrogen). Antifade Gel/Mount aqueous mounting media (Southern Biotech, AL) was applied prior to viewing. Control sections were incubated in PBS instead of primary antibodies to confirm the specificity of immunofluorescence staining. Micrographs of three fields of vision on each section were taken at 200× magnification. The GFAP-, GLT-1-, and CD68-immunoreactive areas were calculated with NIH ImageJ software, and cells expressing caspase-3, NF-200, tau, caspase-3, TDP-43, APP, and GSK3βwere counted within each image.

### Electron microscopy (EM)

Tissues were processed for EM examination as described previously [17]. EM images of various parts of the brain cortex and lumbar spinal cord were captured first at low magnification and then at a higher magnification.

### ROS production

Five rats per treatment group were decapitated 8 weeks after L-BMAA injection. The spinal cord was excised. Oxidative stress was assayed by the 2’,7’-dichlorodihydrofluorescein diacetate (DCFDA) method. A suspension of spinal tissue (200 µl) with a density of 1×10^6^ cells was prepared, rinsed in PBS and stained with 10 µM DCFDA in PBS (Sigma-Aldrich). The suspension was incubated at 37°C for 30 min in darkness and rinsed twice in PBS. A suspension of DCFDA-loaded cells was prepared in 200 µl PBS. The intensity of ROS-dependent fluorescence was recorded at an excitation wavelength of 488 nm and an emission wavelength of 530 nm on a FACS Calibur (BIO-TEK Instruments, Inc., Winooski, VT). The geometric mean of the DCFDA-dependent fluorescence was calculated [41].

### Measurement of interleukin-6 levels

Peripheral blood samples were taken from rats killed by decapitation 2, 4, and 8 weeks after L-BMAA injection (n=3 per group per time point; from the same rats used for Western blot analysis). An enzyme-linked immunosorbent assay for interleukin-6 (Abcam) was carried out as described previously. Absorbance at 450 nm was recorded, and the results were analyzed using GraphPad Prism 4 (GraphPad Software, Inc., San Diego, CA).

### Statistical analysis

All statistical analyses of data to identify the overall effects of treatment were performed with SPSS 13.0 (SPSS, Inc., Chicago, IL). One-way analysis of variance (ANOVA) was carried out on each set of data to ascertain if the differences between groups receiving different treatments were statistically significant. Newman–Keuls post-hoc test and Tukey’s test were then applied. Data are presented as mean ± standard deviation (SD), unless otherwise noted. A p-value<0.05 indicated a statistically significant difference. Kruskal-Wallis nonparametric one-way ANOVA was applied for data obtained as percentages. The results of the rotarod and inclined plate tests were analyzed using Dunnett's test and Steel's test, respectively. GraphPad Prism 4 software was used to prepare the graphs.
